# Laminoplasty plate design is an independent risk factor for facet joint violation

**DOI:** 10.1016/j.jor.2025.05.043

**Published:** 2025-05-26

**Authors:** Alejandro Perez-Albela, Ishan Shah, Tucker Callanan, Timothy Jeng, Sonia Sheth, Bryce Basques

**Affiliations:** a Department of Orthopedic Surgery, Warren Alpert Medical School, Brown University, Providence, RI, USA; b The Warren Alpert Medical School, Brown University, Providence, RI, USA

**Keywords:** Cervical laminoplasty, Facet joint violation, Screw orientation, Laminoplasty plates, Postoperative outcomes, Radiographic outcomes

## Abstract

**Background:**

Cervical laminoplasty is a motion-preserving surgical technique for cervical myelopathy. Laminoplasty plates stabilize the spinal canal, often using different plate designs that define screw trajectory. Although it is recognized that screws penetrating facet joints can lead to worse postoperative pain scores and outcomes, limited research has examined the outcomes associated with the different orientations of laminoplasty plates, which may contribute to screw-related complications such as facet joint violation (FJV). This study evaluates the impact of laminoplasty plate design and screw orientation on FJV incidence, operative time, and clinical outcomes.

**Methods:**

This is a retrospective review of 105 patients receiving unilateral open-door cervical laminoplasty for myelopathy or myeloradiculopathy. Data was collected from 2017 to 2023 at a single institution and procedures were performed by one of six fellowship-trained spine surgeons. Patients with ≥1-year radiographic and clinical follow-up were included. Radiographic parameters (C2–7 Cobb angle, T1 slope, Torg–Pavlov ratio, and C2–7 SVA) were assessed preoperatively, postoperatively, and at 1 year. Groups were separated by the plate design, with one group having horizontal oriented lateral screws and the other having vertical. FJV was defined as screw penetration ≥2 mm beyond the facet cortex.

**Results:**

A total of 105 patients were included (65 horizontal, 40 vertical). Groups were similar in age, BMI, sex, smoking status, diabetes, and CCI (all p > 0.05). Preoperative cervical sagittal parameters were also comparable. Postoperatively, the vertical group had significantly greater loss of C2–7 lordosis (3.22° ± 10.37) compared to the horizontal group (−1.39° ± 11.76; p = 0.03), with this difference persisting at final follow-up (1.38° ± 12.27 vs. −5.21° ± 12.07; p = 0.01). Facet joint violation occurred more frequently in the vertical group (60.0 % vs. 32.3), with multivariate analysis identifying vertical plate orientation as an independent risk factor (OR 11.7, p = 0.01). Vertical plate orientation was also independently associated with increased operative time (+29.5 min, p = 0.02).

**Conclusion:**

Vertical laminoplasty plate orientation was associated with increased FJV risk and longer operative time, as well as greater loss of lordosis within a year postoperatively, though it did not affect pain scores. Further research is needed to evaluate the long-term implications of FJV on patient outcomes.

## 1 Introduction

Cervical laminoplasty is a widely utilized surgical procedure aimed at treating cervical myelopathy while preserving motion. It has become a popular method in cervical myelopathy due to its ability to decompress the spinal cord by opening and lifting the lamina in a hinge-like fashion, effectively enlarging the spinal canal.^1^ Unlike laminectomy, which removes the lamina and may lead to spinal instability, laminoplasty preserves the structural integrity of the spine, maintaining motion and reducing the risk of postoperative kyphotic deformity.^2^ Laminoplasty also offers advantages over posterior cervical decompression and fusion (PCDF), including decreased blood loss, shorter hospital stays, higher rates of home discharge, less morbidity, and is motion-preserving while maintaining similar outcomes for pain, functional status, quality of life, and satisfaction.^3^^,^^4^

Several modifications to the laminoplasty technique have been introduced to optimize surgical outcomes and minimize complications. These include the use of sutures, spacers, and the adoption of laminoplasty plates, which have become the most popular method for stabilizing the lamina after surgery.^5^, ^6^, ^7^ Laminoplasty plates offer controlled and stable expansion of the spinal canal and have been demonstrated to maintain spinal alignment while reducing the risk of laminar reclosure.^8^ However, it is unclear if the placement and orientation of screws within these plates affects surgical outcomes, particularly regarding screw-related complications such as facet joint violation (FJV).^9^

FJV is a relatively common complication in laminoplasty, arising from the close proximity of screw placement to the facet joints, which may inadvertently lead to penetration of these joints.^10^ This may result in postoperative morbidity, including increased axial pain, altered spinal biomechanics, and decreased cervical range of motion.^11^ The reported incidence of FJV related to laminoplasty plate screws varies widely, with studies suggesting rates between 34.1 % and 37.4 %.^12^ These notably high rates of FJV and its potential to compromise clinical outcomes emphasizes the need for further characterization of plate design on outcomes post laminoplasty.^12^

While studies have explored optimal screw angles, entry points, and preventive strategies, there is limited comparative data based on the orientation of the screws in laminoplasty plates—whether horizontal or vertical.^13^, ^14^, ^15^, ^16^, ^17^, ^18^ This study aims to address this gap by determining whether a specific plate design reduces the incidence of screw-related complications, particularly FJV, and improves postoperative outcomes such as pain relief, spinal stability, and range of motion.

## 2 Methods/materials

This study is a retrospective review of a prospective database of patients who underwent unilateral open-door posterior cervical laminoplasty for myelopathy or myeloradiculopathy between 2017 and 2023. These procedures were performed by six fellowship-trained spine surgeons at a single institution. All patients included in the study had a minimum of 1-year radiographic and clinical follow-up. Patients were excluded if they were presenting for sudden trauma, oncologic pathology, or had missing or incomplete data through one year.

Radiographic measurements were taken preoperative, immediate post-op, and at 1 year post-op. These parameters included C2-7 cobb angle, T1 slope, Torg-Pavlov ratio, and C2-7 sagittal vertical axis (SVA). Lordotic cervical angle were reported as negative values and kyphotic cervical angles were reported as positive. Cervical pain was measured using the visual analogue scale (VAS) score and was recorded preoperatively, at 2 weeks, 3 months, 6 months, and 1 year post-op. For further analysis, patients were reclassified into two groups based on the presence or absence of FJV, regardless of plate orientation, and compared across the same radiographic and VAS timepoints. Additionally, outcomes collected included screw backout, plate migration, reoperation rates, C5 palsy, and operation time.

**Fig. 1:**
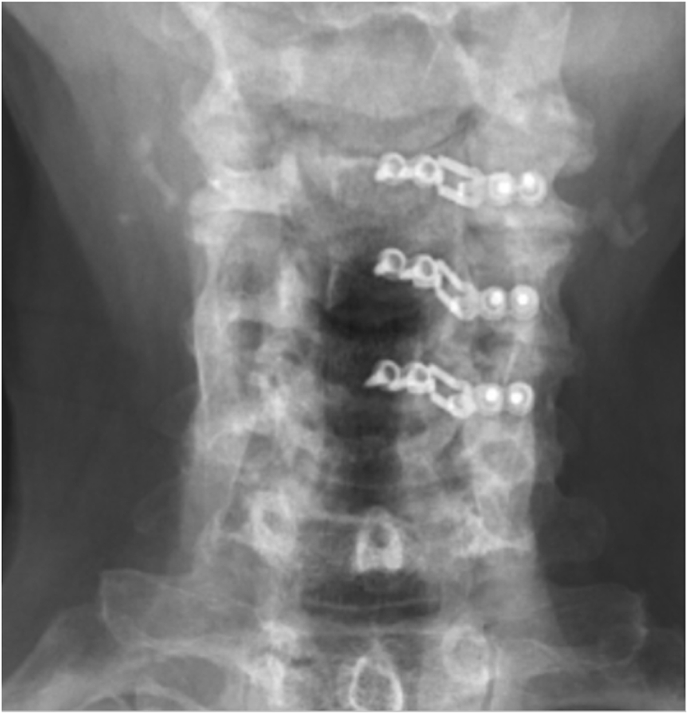
Horizontal laminoplasty plate orientation.

**Fig. 2:**
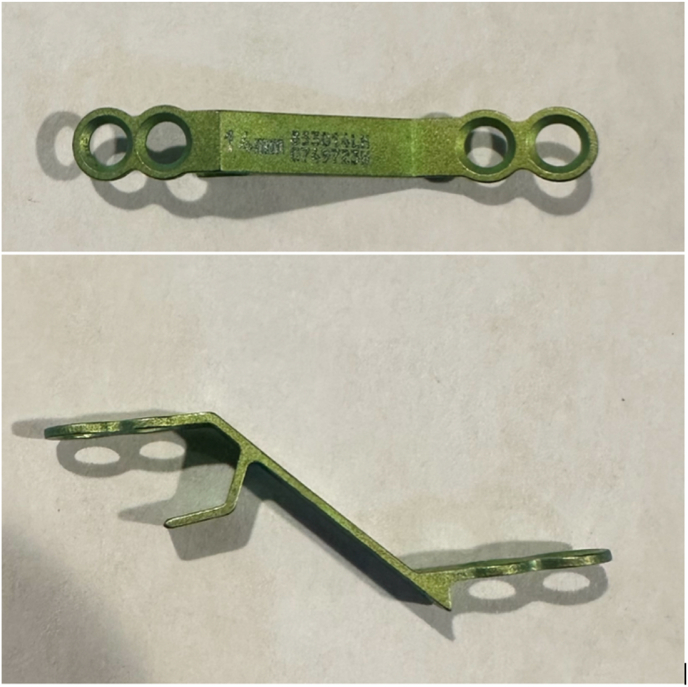
Horizontal oriented laminoplasty plate.

**Fig. 3:**
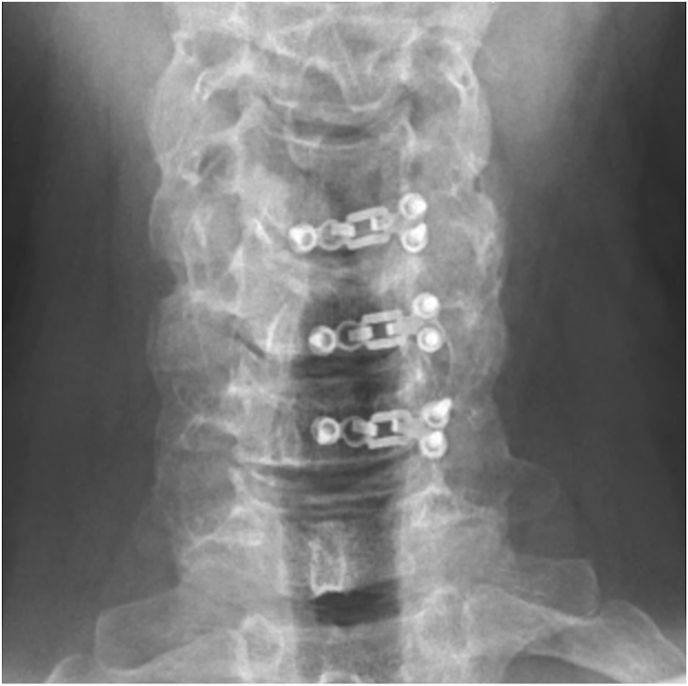
Vertical laminoplasty plate orientation.

**Fig. 4:**
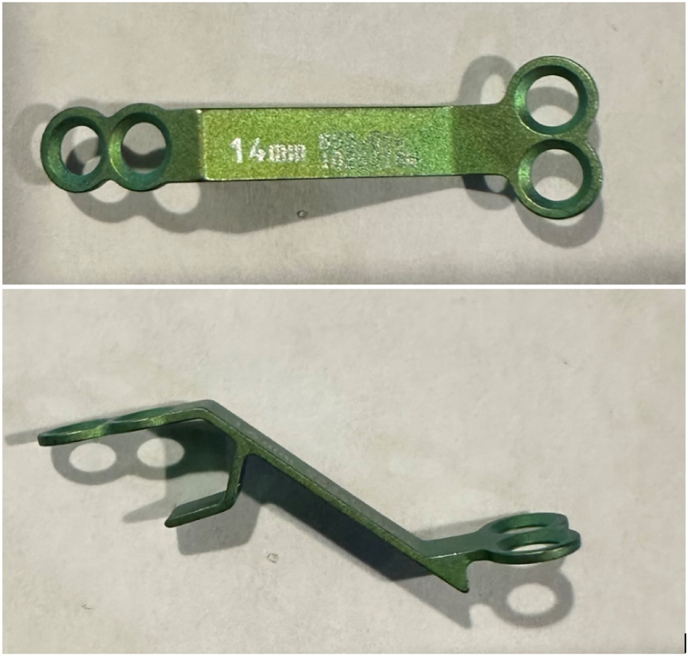
Vertical oriented laminoplasty plate.

Laminoplasty plates were categorized based on the orientation of the lateral screws; horizontal (Fig. 1, Fig. 2) or vertical (Fig. 3, Fig. 4). FJV was defined as any screw penetrating 2 mm or more beyond the outer cortical margin of the facet joint, assessed using lateral cervical x-rays taken within two weeks of the operation. Radiographs were obtained using standardized standing lateral views and assessed via the Ambra Health PACS platform with digital magnification. These measurements were assessed independently by two reviewers and consensus was reached for disagreements by a third reviewer.

Student-t tests was used to compare VAS scores and cervical parameters between groups at defined intervals. Stepwise multivariate regression was performed to determine the independent association of plate lateral screw orientation with the above radiographic parameters and complications. Regression analysis controlled for age, BMI, CCI, smoking status, diabetes status, and screw length.

## 3 Surgical technique

A longitudinal incision was made spanning the operative levels. Dissection proceeded through the fascia and was performed subperiosteally to expose the lamina-facet junction. Care was taken to avoid disruption of soft tissue attachments to C2 and C7. Interspinous tissues were cleared, and the ligamentum flavum was resected using a nerve hook and Kerrison rongeur. Troughs were created bilaterally along the lamina-facet junction at the designated levels, and the lamina at C3 was removed in a lobster-tail fashion. Residual ligamentum flavum was excised. The laminoplasty door was opened by releasing the ligamentum flavum in the lateral gutter and thinning the contralateral lamina. Plates were trialed then secured at the laminoplasty levels with lateral mass and lamina screws, ranging from 6 to 10 mm in length, ensuring stability. Dome laminectomy was performed at the cranial aspect of the caudal lamina as indicated based on stenosis. Closure was performed in layers, with careful reapproximation of the deep muscle and fascia.

## 4 Results

A total of 105 patients were included in the study: 65 in the horizontal plate group and 40 in the vertical plate group. The mean age was 66.4 ± 9.7 years in the horizontal group and 63.7 ± 10.6 years in the vertical group (p = 0.18). Mean BMI was 29.1 ± 6.5 in the horizontal group and 31.8 ± 7.8 in the vertical group (p = 0.07). There were 27 females (41.5 %) in the horizontal group and 11 females (27.5 %) in the vertical group (p = 0.15). Smoking history was reported in 11 patients (17.2 %) in the horizontal group and 2 patients (5.0 %) in the vertical group (p = 0.07). Diabetes was present in 24 patients (36.9 %) in the horizontal group and 10 patients (25.0 %) in the vertical group (p = 0.21). Additionally, there were no differences in CCI between the two groups (p = 0.41) (Table 1).

**Table 1:** Demographics.

Demographics	Horizontal Plate Group (SD)	Vertical Plate Group (SD)	All patients	P-value
Overall (n)	65	40	105	
Age	66.4 ± 9.7	63.7 ± 10.6	65.1 ± 10.2	0.18
BMI	29.1 ± 6.5	31.8 ± 7.8	30.2 ± 7.1	0.07
Female sex	41.54 %	27.50 %	36.19 %	0.15
Smoking	17.19 %	5.00 %	12.50 %	0.07
Diabetes	36.92 %	25.00 %	32.38 %	0.21
CCI 0-2	13.85 %	20.00 %	16.19 %	0.41
CCI 3-4	43.08 %	52.50 %	46.67 %	
CCI 5+	43.08 %	25.00 %	36.19 %	

Preoperatively, no significant differences were noted in cervical sagittal parameters between groups. However, the vertical plate group showed significantly different loss of C2-C7 lordosis postoperatively (3.22° ± 10.37) compared to the horizontal plate group (−1.39° ± 11.76) (p = 0.03), with this difference persisting at final follow-up (1.38° ± 12.27 vs −5.21° ± 12.07) (p = 0.01) (Table 2). There were no significant differences between the horizontal and vertical plate groups in C2–7 SVA, T1 slope, or Torg–Pavlov ratio at any time point. Preoperative, postoperative, and final C2–7 SVA measurements were similar (p = 0.07, 0.44, and 0.61, respectively). T1 slope values did not differ between groups preoperatively (p = 0.21), postoperatively (p = 0.86), or at final follow-up (p = 0.25). Torg–Pavlov ratios were also comparable at all time points (p = 0.67, 0.10, and 0.40). The comparison between patients with and without facet joint violation (FJV) showed no statistically significant differences in any radiographic parameter at preoperative, postoperative, or final follow-up timepoints (Table 3).

**Table 2:** Cervical parameters.

	Horizontal Plate Group Mean (SD)	Vertical Plate Group Mean (SD)	P-value
C2-7 Cobb Angle			
Preoperative	−11.74 ± 11.47	−9.97 ± 8.11	0.35
Postoperative	−1.39 ± 11.76	3.22 ± 10.37	**0.03**
Final	−5.21 ± 12.07	1.38 ± 12.27	**0.01**
**C2-7 SVA**			
Preoperative	2.31 ± 1.06	1.98 ± 0.84	0.07
Postoperative	2.24 ± 1.23	2.15 ± 0.86	0.44
Final	2.84 ± 3.38	2.49 ± 3.70	0.61
**T1 Slope**			
Preoperative	29.91 ± 9.50	27.85 ± 7.49	0.21
Postoperative	28.13 ± 8.35	26.94 ± 5.76	0.86
Final	29.96 ± 9.75	28.20 ± 6.07	0.25
**Torg-Pavlov Ratio**			
Preoperative	0.73 ± 0.11	0.74 ± 0.13	0.67
Postoperative	0.94 ± 0.20	0.88 ± 0.18	0.10
Final	0.91 ± 0.20	0.88 ± 0.17	0.40

Negative cobb angle values indicate lordotic measurements. Positive values are kyphotic measurements.

Sagittal Vertical Axis (SVA).

**Table 3:** Combined facet joint violation radiographic comparison.

	No FJV Mean ± SD	FJV Mean ± SD	P-value
C2–7 Cobb Angle			
Preoperative	−10.74 ± 10.42	−10.93 ± 10.05	0.93
Postoperative	0.22 ± 11.34	0.37 ± 11.05	0.95
Final	−2.33 ± 11.84	−2.70 ± 13.33	0.89
C2-7 SVA			
Preoperative	2.15 ± 1.02	2.22 ± 0.97	0.72
Postoperative	2.11 ± 1.05	2.14 ± 1.12	0.90
Final	2.34 ± 1.02	2.22 ± 1.11	0.61
T1 Slope			
Preoperative	28.97 ± 8.49	29.43 ± 9.44	0.81
Postoperative	27.78 ± 7.90	25.25 ± 6.65	0.16
Final	29.54 ± 8.16	29.10 ± 9.34	0.84
Torg–Pavlov Ratio			
Preoperative	0.73 ± 0.11	0.74 ± 0.12	0.52
Postoperative	0.94 ± 0.19	0.89 ± 0.19	0.23
Final	0.91 ± 0.19	0.89 ± 0.19	0.71

Negative cobb angle values indicate lordotic measurements. Positive values are kyphotic measurements.

Sagittal Vertical Axis (SVA).

**Fig. 5:**
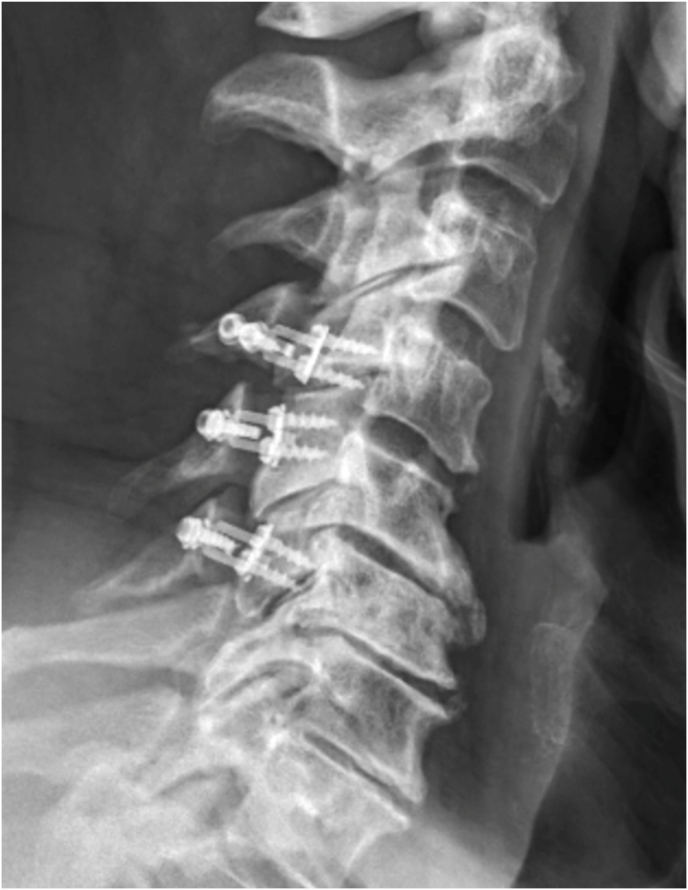
Vertical plate orientation leading to FJV

**Fig. 6:**
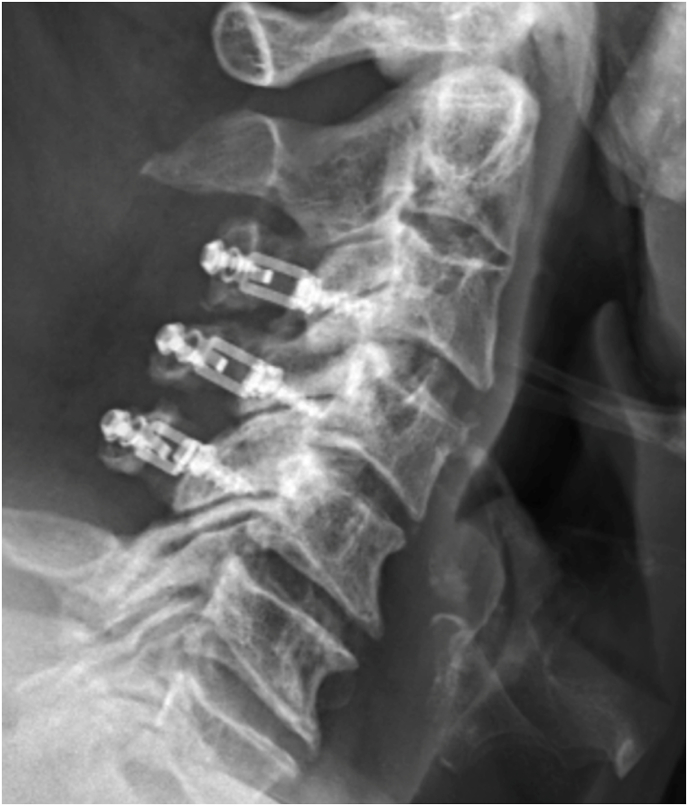
Horizontal plate orientation leading to FJV

A significant difference was observed in the incidence of facet joint screw violation, which occurred in 42.9 % of patients overall (Fig. 5, Fig. 6). This complication was more common in the vertical plate group, with 24/40 patients (60 %) compared to the horizontal plate groups 21/65 patients (32.3 %) (p-value <0.001). Stepwise multivariate logistic regression identified the vertical plate group as an independent risk factor for facet joint violation (OR 11.7, p = 0.001) (Table 4). There was no reported plate migration or failure in this cohort. Screw backout was reported for 2/65 patients (3.1 %) in the horizontal group and 1/40 patients (2.5 %) in the vertical group. Reoperation rates were also 2/65 patients (3.1 %) in the horizontal group and 1/40 patients (2.5 %) in the vertical group. Both groups had a single case of C5 palsy. Multivariate analysis did not find plate orientation to be significantly associated with screw backout, reoperation, or C5 palsy rates. Further multivariate analysis revealed that vertical plate orientation was an independent risk factor for increased OR time, resulting in a mean increase of 29.5 min (p = 0.02).

**Table 4:** Multivariate analysis.

Outcome Variable	Comparison Result	P-value
Operating Room Time	Vertical Group Mean Increase of 29.5 Minutes	**0.02**
Facet Breach	Vertical Group Odds Ratio (OR) 11.7	**<0.01**
Reoperation	Not statistically different	0.58
Screw Backout	Not statistically different	0.65

Preoperatively, the mean Visual Analog Scale (VAS) scores were comparable between the horizontal and vertical plate groups (p = 0.16). Both groups also demonstrated comparable improvement at defined timepoints (Table 5). Both groups demonstrated significant improvements in VAS scores from baseline at each follow-up (2 weeks, 3 months, 6 months, and 1 year), with no significant differences between the two groups at any time point (p > 0.05) (Table 6). There were no significant differences in VAS pain scores between patients with and without facet joint violation (FJV) at any timepoint from preoperative through 1-year postoperative follow-up (Table 7).

**Table 5:** VAS neck scores comparison.

Time Point	Horizontal Orientation: Mean (SD)	Vertical Orientation: Mean (SD)	P-value
Preoperative	5.83 ± 2.58	4.59 ± 2.59	0.16
2-Weeks Postop	3.48 ± 2.11	3.91 ± 2.59	0.6
3-Months Postop	3.04 ± 2.63	2.79 ± 2.94	0.63
6-Months Postop	2.50 ± 2.23	2.72 ± 2.41	0.77
1-Year Postop	2.66 ± 2.27	3.08 ± 2.97	0.78

**Table 6:** Delta VAS score.

Time Point	Horizontal – VAS Delta	P-value	Vertical – VAS Delta	P-value
2-Weeks Postop	1.76	<0.05	1.27	<0.05
3-Months Postop	2.3	<0.05	2.64	<0.05
6-Months Postop	3.25	<0.05	2.72	<0.05
1-Year Postop	2.25	<0.05	1.83	<0.05

**Table 7:** Combined facet joint violation VAS comparison.

Time Point	No FJV Mean (SD)	FJV Mean (SD)	P-value
Preoperative	5.68 ± 2.60	5.36 ± 2.45	0.59
2-Weeks Postop	3.95 ± 2.33	3.69 ± 2.24	0.73
3-Months Postop	2.65 ± 2.62	2.81 ± 2.89	0.85
6-Months Postop	2.14 ± 2.31	2.53 ± 2.32	0.59
1-Year Postop	2.29 ± 2.58	2.71 ± 2.69	0.64

Inter-observer reliability for the assessment of facet joint violation demonstrated strong agreement, with a Cohen's κ of 0.81 (95 % CI: 0.73–0.89). Discrepancies between reviewers were observed in 12 of the 105 cases (11.4 %). These cases were adjudicated by a third senior reviewer to reach a consensus.

## 5 Discussion

Facet joint violation is a recognized complication in cervical laminoplasty, and its occurrence has raised concerns regarding postoperative morbidity, including increased axial pain and altered spinal biomechanics.^10^^,^^11^ Previous studies have looked into screw trajectory to minimize this risk, but the influence of screw hole design in laminoplasty plates remains unclear.^9^ This study evaluated the impact of laminoplasty screw orientation on the incidence of FJV and other associated clinical outcomes. Our results indicate that vertical screw orientation is an independent risk factor for FJV, with a significantly higher incidence in the vertical group (60 %) compared to the horizontal group (32.31 %), with an associated increase in operative time by 29.5 min (p = 0.019). Furthermore, the vertical group and greater loss of cervical lordosis at one year. Despite this, laminoplasty plate design was not associated with reoperation, screw backout, or pain scores at one year post-op.

FJV is a known technical error in cervical procedures that may predispose patients to postoperative pain and increased rates of adjacent segment degeneration.^17^, ^18^, ^19^ Furthermore, previous studies have identified FJV to be associated with negative outcomes such as spinal biomechanics and decreased cervical range of motion.^18^^,^^19^ In this study, The higher FJV rate with vertical screw orientation suggests it more often leads to facet joint encroachment. This may, in part, be influenced by the specific cervical level at which plate orientation is utilized. Cervical facet joints vary in orientation and morphology throughout the spine, with upper cervical facets (e.g., C2–C3) are more coronally oriented, while lower cervical levels (e.g., C5–C7) transition to a more sagittal plane alignment. These anatomical differences influence the location and trajectory required to avoid cortical breach. Although no differences were noted per level in our current study, vertical screws, by their trajectory, may intersect more directly with the sagittal oriented lower cervical facets, increasing the likelihood of violation at these levels. Future, larger studies should evaluate plate design and screw orientation at specific cervical levels to better determine the optimal configuration that minimizes complications such as FJV.

Another notable finding from our study is the increased operative time associated with the use of vertical screws. The vertical plate group experienced an average increase of 29.5 min in operative time. This may reflect the additional time required to avoid FJV during screw placement in this configuration. However, despite this increased time, there was still a higher rate of FJV in the vertical cohort.^20^, ^21^, ^22^ This paradox may also be explained by inherent technical challenges of aligning vertical screws parallel to the lateral mass surface without breaching the facet, particularly at lower cervical levels. Even with additional time taken for careful placement, the trajectory required for vertical screws at certain levels may be at greater risk for cortical breach. Furthermore, longer operative times raise concerns about the potential for increased perioperative complications, including those related to prolonged anesthesia exposure, such as higher risks of cardiovascular events, infection rates, and delayed recovery.^21^^,^^22^

The consistent reduction in Visual Analog Scale (VAS) scores across both horizontal and vertical plate orientations suggests that lateral screw orientation did not significantly alter clinical outcomes within one year. Both groups exhibited neck pain improvement by 1 year, and facet joint violation was not associated with neck pain in this study. Although prior studies have linked facet breach to worse outcomes, this relationship is not strictly linear—some patients with facet violation experience no pain, while others without violation may report significant symptoms, which may have conflated our outcomes. Otherwise, patients in the vertical group did have greater loss of lordosis over the course of a year, and this kyphotic cervical alignment could be a compensation mechanism for the facet violation that leads to undetectable differences in pain scores. It is also possible that soft tissue or muscular compensation may initially buffer the biomechanical effects of facet violation, delaying symptom onset. Alternatively, it is also possible that differences related to FJV may emerge over longer follow-up periods and were not detectable within the one-year timeframe of this study. This may parallel findings in lumbar spine surgery, where FJV did not always correlate with immediate clinical deterioration but posed a risk for future adjacent segment disease and other long-term complications.^19^ Overall, despite the higher rate of FJV in the vertical plate group, this did not translate into reduced patient satisfaction at one year.^23^^,^^24^ However, the long-term impact of FJV on reoperation rates, spinal alignment, and patient satisfaction warrants further investigation.

There are several limitations to this study that should be considered when interpreting the results. First, as a retrospective review of a prospective database, there is inherent potential for selection bias. Moreover, our follow-up period was one year to allow enough clinical and radiographic follow up. While this time frame allows us to capture shorter-term outcomes, it may not be sufficient to evaluate the long-term effects of FJV, such as adjacent segment disease, persistent axial pain, or degenerative changes. Additionally, myelopathy outcome scores such as mJOA were not routinely captured for enough patients to be used in analysis. However as this study was designed to assess concerns related to FJV due to plate design, assessment of myelopathy outcomes was outside the scope of this study and likely would not affect conclusions. Furthermore, VAS was the only available PRO, and the lack of other scores such as the Neck Disability Index (NDI) limits the comprehensiveness of our clinical outcome assessment. Finally, the assessment of FJV was based on standard postoperative cervical X-rays, which may lack the sensitivity to detect subtle violations, although the incidence of FJV identified in this study was similar to prior studies. Suboptimal cervical X-rays or patient positioning can also contribute to misinterpretation of FJV. Computed tomography (CT) would likely capture FJV more accurately, however CT scans are not a routine part of the postoperative protocol for laminoplasty patients, and adding this test may expose patients to additional radiation without clear clinical benefit. Furthermore to mitigate this limitation, two independent reviewers read films to ensure accuracy.

## 6 Conclusion

Vertical orientation of lateral screws in laminoplasty plates is associated with significantly higher mean operative time and rate of facet joint violation, with greater loss of cervical lordosis at one year compared to horizontal plates. However, orientation was not associated other post-operative cervical parameters, neck VAS scores, screw backout, or reoperation rates.

## CRediT authorship contribution statement

**Alejandro Perez-Albela:** Methodology, Formal analysis, Writing – original draft, Visualization, Project administration. **Ishan Shah:** Data curation, Investigation. **Tucker Callanan:** Writing – review & editing. **Timothy Jeng:** Data curation, Investigation. **Sonia Sheth:** Data curation. **Bryce Basques:** Conceptualization, Supervision, Methodology, Writing – review & editing.

## Ethical statement

This study was approved by the Lifespan Institutional Review Board (IRB 2), under protocol number **1826708**, with initial approval granted on **March 14, 2022**. The project was deemed minimal risk and qualified for expedited review under **45 CFR 46.110(b)(1), Category 5**. A general waiver of informed consent and HIPAA authorization was granted in accordance with **45 CFR 46.116(f)(1)**. All procedures were performed in accordance with institutional guidelines and the ethical standards outlined in the Declaration of Helsinki and its amendments. Patient confidentiality was maintained throughout the study, and no animals or transplanted tissues were involved.

## Funding

None.

## Declaration of interest:

The authors declare the following financial interests and personal relationships that may be considered potential competing interests: BB reports consulting for Medtronic, Globus, and Stryker. All other authors declare no disclosures.
